# Clear Cell Renal Cell Carcinoma Spinal Metastases: Which Factors Matter to the Overall Survival? A 10-Year Experience of a High-Volume Tumor Spine Center

**DOI:** 10.3390/diagnostics12102442

**Published:** 2022-10-09

**Authors:** Silvia Terzi, Valerio Pipola, Cristiana Griffoni, Federica Trentin, Elisa Carretta, Annalisa Monetta, Fabio Vita, Stefano Bandiera, Giovanni Barbanti-Bròdano, Riccardo Ghermandi, Gisberto Evangelisti, Giuseppe Tedesco, Marco Girolami, Carlotta Cavallari, Alessandro Gasbarrini

**Affiliations:** 1 Department of Spine Surgery, IRCCS Istituto Ortopedico Rizzoli, 40136 Bologna, Italy; 2 Department of Programming and Monitoring, IRCCS Istituto Ortopedico Rizzoli, 40136 Bologna, Italy

**Keywords:** cear cell renal cell carcinoma, spinal metastases, en-bloc resection, visceral metastases, Tokuhashi score

## Abstract

Clear cell renal cell carcinoma (ccRCC) usually spreads in the spinal region causing instability or spinal cord compression leading to neurological deficits. Therefore, surgical treatment is required for improving the outcome of patients. The aim of this study is to identify which prognostic factors could affect overall survival in patients affected by ccRCC. **Methods:** Retrospective cohort study of patients with ccRCC spinal metastases, surgically treated from November 2009 to April 2019. Demographic and clinical data were collected. The Kaplan–Meier method was used to estimate overall survival, and the log-rank test was used to evaluate differences in survival among potentially prognostic factors. **Results:** A total of 69 patients were surgically treated and followed up for a median period of 65 months. The average age at the time of surgery was 62.6 years old. The median overall survival (OS) was 34.7 months (95% CI 20.8–51.9) and 5-year OS was 31.2% (95% CI 19.2–44.1). A high Tokuhashi score (*p* = 0.0217), the presence of visceral metastases (*p* < 0.001), other bone metastases (*p* = 0.02012) and the kind of surgical treatment (*p* = 0.0395) are the main prognostic factors that influence the OS. Moreover, 3-year progression-free survival (PFS) was analyzed: the median PFS was 53.1 months and the % 3-year PFS was 62.9% (45.2–76.3). In the multivariate analysis, only pre-operative radiation therapy had a significant impact on 3-year PFS (95% CI 0.929–12.994, *p* = 0.0643). **Conclusion:** The results of this study suggest that the absence of visceral metastases and an aggressive surgery as en-bloc, when feasible, could prolong the survival rate and improve quality of life for patients.

## 1. Introduction

Clear Cell Renal Cell Carcinoma (ccRCC) accounts for 3% of all adult malignancies [1,2]. Approximately 25% of patients with ccRCC present with locally advanced or metastatic disease at diagnosis.

Following the lung, bone is the most common site of metastatic disease, with spine involved in 40% of bony metastases [3,4]. The lesions are predominantly osteolytic, and the clinical picture is characterized by varying degrees by pain, instability and neurological deficit.

To choose the best treatment, several factors must be taken into account. It has to be individualized and multidisciplinary. The surgeon’s intention is more often palliative, with the aim of improving or maintaining the quality of life, affecting survival only indirectly. In the past decade, a significant improvement has been reported in the systemic management of ccRCC with the introduction of immunotherapy and target therapy, loading the prolongation of survival, even in the case of metastatic disease [5,6,7,8].

Surgery, preceded by embolization, is still the most used and proven technique, especially in the case of instability or MSCC (metastatic spinal cord compression) [9]. However, it is necessary to consider other emerging techniques, such as stereotactic-radiotherapy, and the need for systemic therapy to avoid the spread of the disease. Concerning the radiation therapy techniques, some adaptations have been introduced in conventional radiotherapy and several protocols have been assessed to modulate the total dose and achieve a better local control, lower rate of re-treatment and lower risk of pathological fracture [10]. However, in particular, new radiation therapy techniques have been developed: stereotactic-radiosurgery (SRS) (one fraction) and stereotactic-body-radiotherapy (SBRT) (two–three fractions). They consist of delivering a high dose, in one–three fractions, in a small target with rapid fall-out of the dose, enabling the radiation dose to be confined more precisely to treatment volume preserving the spinal cord or other critical structures [11]. Nevertheless, ccRCC has shown to be poorly responsive to radiotherapy and systemic therapy; consequently, surgery for spinal metastases of ccRCC is the lead actor, having proved to be prominent in the performance of a curative intent [12] even with aggressive interventions of en-bloc removal.

The present study describes the clinical experience gained from 2009 to 2019 within a department dedicated to vertebral oncological surgery. The aim of this study is to assess the overall survival of surgically treated patients with ccRCC spinal metastases and to identify which prognostic factors could be related to a better survival rate.

## 2. Materials and Methods

Retrospective observational cohort study of consecutive patients surgically treated for spinal ccRCC metastases at a high-volume tumor spine center from November 2009 to April 2019. The study has been approved by the Emilia Romagna Ethics Committee (prot. number 0007434 of 21/06/2018).

Demographic, anamnestic and clinical data have been extracted from electronic health records. For each patient at baseline, we assessed the localization of vertebral metastases, the presence of other bone or visceral metastases, the neurological status according to Frankel score [13], the ambulatory autonomy and the general status measured with the Karnofsky Performance Scale (KPS) [14]. The expected prognosis was retrospectively assessed according to the revised Tokuhashi score (TSS) [15]. Each therapeutic decision followed the multidisciplinary evaluation of a team composed of oncologist, radiotherapist and spine surgeon.

We considered the time interval between the diagnosis of primary ccRCC and the occurrence of spinal metastases. Bony or visceral metastases were defined as synchronous when detected at the same time of primary tumor diagnosis.

Data regarding non-surgical therapies (radiotherapy and systemic therapies) performed before and after surgery; intra- and post-operative complications; any re-operations and local recurrence of the disease were collected.

The same clinical aspects seen at baseline (progression of systemic disease, Frankel score, Karnofsky Performance Scale and ambulatory status) were evaluated at follow-up visits. For all the patients who were lost at follow-up and could not be directly contacted, a search was made at the local Health Inspector, in order to obtain information about if and when the death took place.

The performed surgical procedures were divided into four categories: minimally invasive surgery (minimally invasive stabilization, thermoablation, vertebroplasty), palliative surgery (decompression associated or not with stabilization), debulking (intended as macroscopically large removal of metastasis with an intralesional margin, associated with stabilization) and en-bloc resection.

Moreover, intra-operative and post-operative complications were evaluated and classified using the Spinal Adverse Events Severity System (SAVES-V2) [16].

### Statistical Analysis

The demographic and clinical characteristics of the study cohort were summarized using mean, median and range or absolute and percentage frequencies. Given the limited number of events and observations in this study, variable categories were grouped when appropriate. Bhapkar’s tests of marginal homogeneity was used to compare clinical score before and after surgery. The outcome of interest was overall survival (OS) defined as the time elapsed from the date of surgery to the date of death or to the date of the last follow-up. The Kaplan–Meier method was used to estimate OS and the log-rank test was calculated to evaluate differences in survival among potentially prognostic factors. Cox regression analysis was used to determine multivariate hazard ratios (HRs) for significant prognostic factor in univariate analysis. A *p* value of <0.05 was considered statistically significant. All analyses were performed using SAS 9.4 (SAS Institute, Cary, NC, USA) software.

## 3. Results

Between November 2009 and April 2019, 69 patients (49 males and 20 females) affected by spinal metastases from clear cell renal cell carcinoma were surgically treated at our center and followed up for a median period of 65 months (IQR 49–99). The average age was 62.6 years old (range 36–82). Twenty-six patients suffered from synchronous metastases and twenty-eight patients had visceral metastases at the time of surgical procedure. Furthermore, twenty-nine patients had other bony metastases. The number and localization of spinal metastases at the surgical time are reported in Table 1, while data concerning the sites of visceral metastases have been reported in Table 1. Data regarding previous treatments received by patients before the surgical treatment received at our center are reported in (Table 1).

Survival prediction and the choice of surgical procedure were evaluated using the Tokuhashi score (Table 1). Patient distribution in the three prognostic groups defined by the Tokuhashi cutoff score was as follows: score 0–8 (predicted survival <6 months) for 17 (24.6%) patients; score 9–11 (predicted survival >6 months) for 35 (50.7%) patients; score 12–15 (predicted survival >12 months) for 12 (17.4%) patients.

Moreover, the Spinal Instability Neoplastic Score (SINS) was used for the evaluation of surgical approach. Of the total number of patients seen, the SINS score was potentially stable in 48 (69.6%) and unstable in 21 (30.4%). However, from the univariate analysis, SINS was an independent factor not correlated with the overall survival (*p* = 0.2735).

Ten patients (14.5%) were treated with en-bloc resection achieved with wide margin in six cases, marginal margin in two cases and an intralesional margin in the other two cases. Fifty patients (72.5%) were submitted to a debulking surgery, seven patients (10.1%) to a palliative surgery (decompression and stabilization without tumor excision) and two patients (2.9%) to minimally invasive stabilization (Table 2).

Twenty-one out of sixty-nine patients (30.4%) had a total of 25 complications. Among patients with complications, 66.7% (14/21) were submitted to a debulking surgery, 23.8% (5/21) were submitted to en-bloc resection and 9.5% (2/21) were submitted to palliative surgery. One of them (paraplegy) occurred during the presurgical embolization. Overall, we detected six intra-operative complications and nineteen post-operative complications. The most common intra-operative complication was a dural tear with a 16% rate (4/25 cases), while the most common post-operative complication was a deep wound infection detected in 28% of cases (7/25) (Table 3).

Data regarding pre- and post-operative ambulatory status and Frankel and Karnofsky scores are reported in Table 4. Overall, significant improvement in ambulatory and neurological status was observed in the cohort after surgery (respectively, *p* < 0.0001 and *p* = 0.0003). More in depth, ambulatory ability (with or without aids) was maintained in all patients who could ambulate before surgery (48/49, 98%), except for one patient that underwent paraplegy after embolization as a complication, and was recovered in 15/20 (75%) patients who lost ambulatory ability before surgery. No significant change in Karnofsky performance status was found after surgery (*p* = 0.2809) (Table 4).

**Figure 1:**
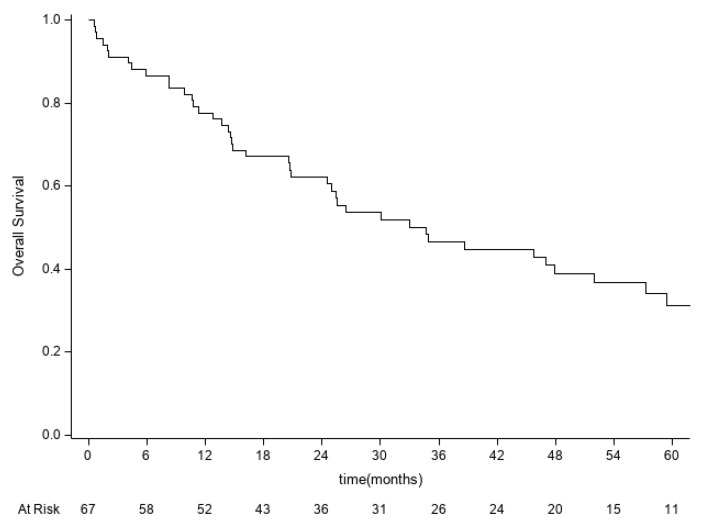
Kaplan–Meier overall survival curve.

The median overall survival was 34.7 months (95% CI 20.8–51.9) and 5-year OS was 31.2% (95% CI 19.2–44.1). Forty-four patients died from disease during the considered observation period (Figure 1).

The univariate analysis conducted for clinical and demographic features, reported in Table 5, suggested that factors capable of influencing overall survival are the Tokuhashi score (*p* = 0.0209), the presence of visceral metastases (*p* < 0.001) and other bony metastases (*p* = 0.0188) and the kind of surgical treatment received (*p* = 0.0395). The 5-year OS was 28% (95% CI 14.8–42.8) for debulking surgery, 67.5% (95% CI 29.1–88.2) for en-bloc resection and 16.7% (95% CI 1.1–49.3) for other palliative treatments. Prior or post-operative chemo- and radiotherapy treatments did not influence the overall survival at the univariate analysis. Results from the multivariate analysis confirmed the presence of visceral metastases as a significant prognostic factor for overall survival (HR (95% CI): 2.5 (1.2–5.3), *p* = 0.0186), whereas no significant association was found for the Tokuhashi score, other bony metastases and the kind of surgical treatment.

**Figure 2:**
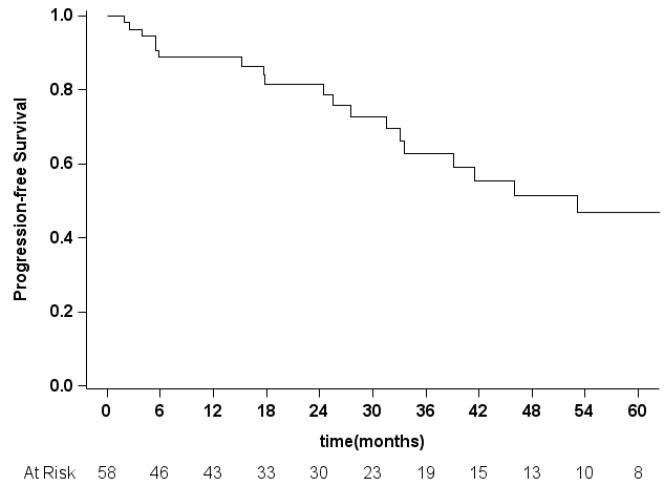
Kaplan–Meier progression-free survival curve.

Due to the relevance of the local control of disease, we also performed a Kaplan–Meier analysis of 3-years progression-free survival (PFS), considering patients having a recurrence at the surgical site. We failed to retrieve data concerning 11 patients, thus we performed the analysis on 58 patients (Figure 2). The median PFS was 53.1 months and the % 3-year PFS was 62.9% (45.2–76.3).

The univariate analysis of prognostic factors for 3-year PFS, reported in Table 6, indicated that it is significantly affected by the presence of other bony metastases (*p* = 0.0210) and pre-operative radiation therapy (*p* = 0.0047). In the multivariate analysis only pre-operative radiation therapy had a significant impact on 3-year PFS (95% CI 0.929–12.994, *p* = 0.0643).

## 4. Discussion

Clear cell renal cell carcinoma (ccRCC) is the most common subtype of renal cell carcinoma (RCC), accounting for approximately 70% of all RCC cases; it derives from the proximal convoluted tubule and is characterized by aberrant angiogenesis. Bone metastases are the second most frequent distant site of metastatic ccRCC. The incidence of bone metastasis ranges now between 15 and 34%. Spine metastasis, a subtype of bone metastasis, is considered a negative prognostic factor [17] as it is a difficult disease to treat. The 5-year survival rate in patients with bone metastasis in the spine is 9%, compared with 30% in the appendicular skeleton [18]. Through hematogenous dissemination or local invasion, the tumor spreads to the spine. The ccRCC has a well-known angiotropism associated with the anatomical and hemodynamic characteristics of the blood supply of the spine and to the persistence of hematopoietic tissue inside the vertebral body, making this region the most susceptible localization for the metastases.

Considering ccRCC metastases, surgical treatment is the only method that can improve the patient’s quality of life. Since our investigation started in 2009 for this cohort of patients, the surgical decision making and the type of surgical procedure have been settled based on the flow chart for the management of spinal metastases validated by Gasbarrini et al. [19,20] and on SINS score for evaluation of spinal instability validated by Fourney et al. [21].

The algorithm we used for the management of spinal metastases starts from the patient’s response to existing systemic and radiotherapy treatments as a fundamental element for surgical decision making. The updating of systemic and radiotherapy treatments has always been taken into consideration thanks to an integrated multidisciplinary approach between spine surgeons, medical oncologists and radiation therapy oncologists.

During the period of our investigation, other frameworks, scores and algorithms have been introduced to guide the management of spinal metastases and they have been recently reviewed [22]. In particular, we integrated our algorithm with the neurologic, oncologic, mechanical and systemic (NOMS) decision framework, reported in 2013 [23].

The results of the present study showed that the type of surgical treatment was associated with different survival rates.

Debulking treatment (intracapsular intralesional excision) was performed in patients who subsequently underwent chemotherapy and radiotherapy to minimize the possibility of local recurrence. This treatment showed a 5-year overall survival of 28%.

En-bloc resection, instead, was performed in patients who had intact single spinal metastases, a good general and neurologic status with Frankel score E. This type of treatment had proved the highest 5-year overall survival value equal to 67.5%.

Surgical palliative treatment and MIS showed a 5-year overall survival of 16.7%. These patients had poor prognosis due to a more severe clinical status with systemic disease progression at the visceral and skeletal levels.

In fact, the progression of systemic disease with multi-metastatic localizations at the visceral and bone levels resulted in being an unfavorable factor for overall survival, as demonstrated in our analysis.

Patients with a solitary bone metastasis had a longer survival than patients with other bone metastases, according to Jianpo Zhai et al. [24].

Moreover, we observed that the absence of visceral metastases is the most significant prognostic factor correlated with prolonged overall survival, as confirmed by the multivariate analysis. Indeed, the median survival time was 14.8 months and 57.2 months, respectively, in patients with visceral metastases and those without visceral metastases. This result is consistent with other reports: Ruatta et al. took a large-scale single-center prognosis study on 300 patients with bone metastases (BM) from ccRCC and found that the OS of patients with visceral metastasis was significantly shorter than OS of patients without visceral metastasis (17.6 months vs 46.4 months, *p* < 0.0001) [25].

Shinohara et al. investigated 50 RCC patients with bone metastasis. The univariate and multivariate Cox regression analyses indicated that visceral metastasis was an independent unfavorable prognostic factor [26].

Patients with visceral metastases have a systemic spread of the disease and their survival depends on this spreading of the disease. Spinal surgery affects survival only if it is curative, and it is curative only in the case of en-bloc resection, which is indicated in the case of isolated metastasis and good general and neurologic status. In other cases, for patients with spinal metastases (with or without visceral metastases), surgery has a palliative and/or functional intent to improve quality of life and restore neurological function and, if possible, it is associated with a local control of the disease.

Moreover, we recently reported that in breast cancer spinal metastases, the presence of concurrent bone metastases is the first adverse prognostic factor affecting survival [27].

Given the poor prognosis of ccRCC patients with visceral metastasis, palliative and mini-invasive surgeries have been the main treatments considering the general status aimed at improving quality of life in the short term after surgery, while more aggressive surgery was indicated in patients without visceral metastases and with a Frankel score D-E and good general status.

We also observed that in patients affected by non-small cell lung cancer (NSCLC) spinal metastases, having a shortened life expectancy, the surgical treatment was able to improve the quality of life by controlling pain and enhancing the neurological status and autonomy [28].

Hence, the most important goal of surgical resection in patients with spinal metastatic disease is to preserve or restore neurological function. Our study shows that the ambulatory capability and the neurological function could be preserved or restored by surgery until recurrence or other spinal cord compression occurred, improving the quality of life of the patient and their self-ability. However, our results showed that the ambulatory status, the Frankel score and Karnofsky Performance Status were not associated with survival rate.

Among the prognostic factors that we examined, patient’s age, previous treatments (such as chemotherapy or radiotherapy) and previous surgeries at the index site do not seem to influence the survival rate.

We also analyzed the 3-year progression-free survival and observed that it was significantly affected by pre-operative radiation therapy. We think that two reasons may explain the greater rate of local recurrence in the presence of pre-operative radiation therapy: the need to use low doses of post-operative radiation therapy at the surgical site if it was already performed before surgery; the presence of tissue damage due to radiotherapy that can limit the surgical removal of the metastasis.

In this retrospective study, adverse events and complications occurring intra-operatively and post-operatively were analyzed.

Complications affecting surgical treatment of spinal metastases have been extensively studied because the occurrence of adverse events can significantly impair the final goal of improving the patient’s quality of life [26,27,28,29,30,31]. In our study, the complication rate was 30.4%, with a total of 25 complications. Five out of twenty-five complications were related to the en-bloc resection treatment: one had pneumothorax as an intra-operative complication, two patients had deep wound dehiscence and one had rods breakage as post-operative complications and one patient had neurologic deterioration (paraplegia) due to presurgical embolization. In agreement with Yao et al. [32] in their systematic review, the en-bloc resection of solitary ccRCC metastases resulted in a low complication rate and prolonged survival. In general, we did not observe any correlation between the presence of surgical complications and survival rate.

The present study analyzes the prognostic factors influencing the overall survival in patients with vertebral metastases of ccRCC. However, some limitations should be acknowledged. These include the small sample size of our cohort, the retrospective data collection which can introduce bias and errors and lack of a control group of renal cancer patients without surgically treated spinal metastases.

## 5. Conclusions

Despite the limits relating to the retrospective analysis carried out and considering the development of the new therapeutic strategies in recent years [33,34,35], this study showed that the main prognostic factor influencing the overall survival of patients with renal cell carcinoma spinal metastases is the presence of visceral metastases. In the absence of visceral metastases, the surgical treatment of spinal metastases is indicated: in particular, en-bloc resection, when feasible in relation to the prognosis assessed by the Tokuhashi score, represents the surgical treatment associated with higher overall survival. In all other cases, the goal of surgery remains to preserve and/or restore neurological function and spinal stability in order to improve the patient’s quality of life.

The increase in survival, related to the introduction of target therapies even in patients with visceral metastases, is inevitably associated with an increase in local disease recurrence rates in patients treated with functional surgery.

In such a scenario, multidisciplinary evaluation with oncologists and radiotherapists remains of primary importance. Procedures such as selective arterial embolization can help promote local disease control [36]. In the case of neurological instability or decline in neurological function, re-intervention should be considered.

## Figures and Tables

**Table 1:** Demographic and clinical data and sites of visceral metastases.

Characteristics	N	%
*Age (years)*		
<65	36	52.2
≥65	33	47.8
*Gender*		
Female	20	29.0
Male	49	71.0
*Intact*		
Yes	9	13.0
No	60	87.0
*Synchronous metastases*		
Yes	26	37.7
No	42	60.9
NA	1	1.4
*Visceral metastases*		
Yes	28	40.6
No	37	53.6
NA	4	5.8
*SINS score*		
7–12	48	69.6
13–18	21	30.4
*Previous Treatment*		
Surgery	23	33.3
Surgery + RT	4	5.8
Surgery + RT + CHT	13	18.9
Surgery + CHT	10	14.5
RT + CHT	7	10.1
CHT	1	1.4
NA	11	16.0
*Tokuhashi score*		
0–8	17	24.6
9–11	35	50.7
12–15	12	17.4
NA	5	7.3
*Sites of visceral metastases*		
Lungs	19	
Spleen	3	
Liver	3	
Thyroid	1	
Esophagus	1	
Uterus	1	
Adrenal gland	3	
Brain	1	

The total number of spinal metastases is 105 because some patients presented more than one vertebra affected by tumor not necessarily treated surgically.

**Table 2:** Surgery data.

	N	%
*Type of surgery*		
MIS	2	2.9
Palliative surgery	7	10.1
Debulking	50	14.5
En-bloc	10	72.5
*Margins*		
Intralesional	61	88.4
Marginal	2	2.9
Wide	6	8.7
*Complications*		
Yes	21	69.6
No	48	30.4

**Table 3:** Intra-operative and post-operative complications.

Intra-Operative		Post-Operative	
	n/N (%)		n/N (%)
Dural tear	4/25 (16%)	Wound dehiscence	3/25 (16%)
Neurological impairment *	1/25 (4%)	Deep wound infection	7/25 (28%)
Other	1/25 (4%)	Superficial wound infection	1/25 (4%)
		Pulmonary embolism	1/25 (4%)
		Construct failure without loss of correction	4/25 (12%)
		CSF leak/meningocele	1/25 (4%)
		Pneumonia	1/25 (4%)
		Other	1/25 (4%)
Total	6/25		19/25

Note: * Paraplegy after embolization, not related to surgical procedure.

**Table 4:** Comparison between pre-operative and post-operative patient status.

	Pre-Operative		Post-Operative		*p* Value
	N	%	N	%	
*Ambulatory status*					<0.0001
Bedridden	17	24.6	3	4.3	
Wheelchair	3	4.3	3	4.3	
Walking aids	8	11.6	35	50.7	
Self-supporting	41	59.4	28	40.6	
*Frankel score*					0.0003
E	42	60.9	56	81.2	
other	27	39.1	13	18.8	
*Karnofsky score*					0.2809
<70	36	52.2	39	56.5	
≥70	32	46.4	28	40.6	
NA	1	1.4	2	2.9	

Note: NA, not available.

**Table 5:** Univariate analysis of the prognostic factors affecting overall survival.

Variables	Median OS, Months (95% CI)	% 5-Year OS (95% CI)	Log-Rank Test *p* Value
*Age*			0.1742
<65	26.5 (12.8–57.2)	25.8% (11.2–43.1)	
≥65	47.0 (24.5–70.0)	37.2% (18.9–55.7)	
*Gender*			0.0712
Female	58.0 (20.8-nr)	42.5% (18.6–64.8)	
Male	25.4 (14.8–38.6)	27.5% (14.4–42.3)	
*SINS score*			
7–12	26.5 (20.5–51.9)	27.5% (14.9–41.7)	0.2735
13–18	47.9 (9.8-nr)	45.0% (18.9–68.2)	
*Pre-operative Ambulatory status*			
Bedridden/Wheelchair/Walking aids	20.7 (10.6–93.1)	28.9% (10.3–50.7)	0.3595
Self-supporting/walking aids	34.9 (25.4–58.0)	32.6% (18.0–48.1)	
*Pre-operative Karnofsky score*			0.5343
<70	26.5 (14.7–47.9)	28.0% (13.4–44.5)	
≥70	38.6 (16.1–70)	34.7% (16.3–54.0)	
*Post-operative Karnofsky score*			
<70	24.5 (14.3–47.9)	32.6% (17.5–48.7)	0.4034
≥70	38.6 (25.4–70)	32.3% (14.3–51.9)	
*Intact*			
No	23.7 (62.3- nr)	33.3% (5.6–65.8)	0.6630
Yes	34.9 (24.5–57.2)	31.6% (18.9–45.1)	
*Pre-operative Frankel score*			
E	34.7 (20.8–58.0)	28.7% (13.5–45.9)	0.7379
other	26.5 (11.3–93.1)	34.2% (16.3–53.0)	
*Post-operative Frankel score*			
E	36.6 (25.0–58.0)	32.7% (19.0–47.0)	0.2084
other	14.6 (1.9-nr)	30.8% (9.5–55.4)	
*Tokuhashi score*			
0–8	14.7 (1.9–38.6)	23.4% (6.5–46.3)	0.0209
9–11	30.1 (16.1–51.9)	22.5% (9.1–39.4)	
12–15	nr	78.6% (36.1–94.4)	
*Visceral Metastases*			
No	57.2 (34.7–112.3)	48.8% (29.8–65.4)	<0.001
Yes	14.8 (8.3–25.0)	9.0% (0.9–29.3)	
*Other metastases*			
No	51.9 (25.4–112.3)	42.0% (23.8–59.2)	0.0188
Yes	19.6 (9.8–47.0)	18.7% (5.8–37.3)	
*Complications*			
No	45.7 (25.4–58.0)	33.7% (19.1–49.0)	0.3632
Yes	24.5 (14.3-nr))	30.6% (12.3–51.2)	
*Surgery types*			
Debulking	33.1 (20.5–51.9)	28.0% (14.8–42.8)	0.0395
En-bloc	nr	67.5% (29.1–88.2)	
other	14.8 (0.7–47.0)	16.7% (1.1–49.3)	
*Pre-radiation*			
No	34.9 (20.8–70.0)	33.4% (17.3–50.3)	0.3591
Yes	25.0 (12.8–93.1)	33.2% (15.8–51.9)	
*Post-radiation*			
No	45.7 (16.1-nr)	39.5% (19.2–59.2)	0.6594
Yes	51.9 (26.5–70)	34.9% (12.5–58.0)	
*Pre-chemotherapy*			
No	45.7 (20.5–70)	32.3% (13.4–53.0)	0.9785
Yes	25.6 (14.7–93.1)	35.8% (17.9–54.1)	
*Post-chemotherapy*			
No	nr	57.1% (17.2–83.7)	0.6257
Yes	47.9 (26.5–93.1)	38.2% (21.0–55.3)	

Note: nr, not reached.

**Table 6:** Univariate analysis of the prognostic factors affecting 3-year progression-free survival (PFS).

	% 3-Year PFS (95% CI)	Log-Rank Test *p* Value
*Age*		0.2452
<65	58.7% (37.1–78.7)	
≥65	65.4% (40.8–86.3)	
*Gender*		0.5787
Female	63.9% (33.3–83.3)	
Male	63.3% (40.9–79.2)	
*SINS score*		
7–12	59.5% (38.4–75.5)	0.3446
13–18	71.8% (34.9–90.0)	
*Pre-operative Ambulatory status*		
Bedridden/Wheelchair/Walking aids	53.1% (20.3–77.6)	0.1347
Self-supporting/walking aids	67.3% (46.4–81.5)	
*Pre-operative Karnofsky score*		0.8091
<70	57.9% (33.7–76.0)	
≥70	67.7% (39.3–84.9)	
*Post-operative Karnofsky score*		
<70	62.8% (36.0–80.9)	0.7643
≥70	63.5% (37.0–81.3)	
*Intact*		
No	37.5% (1.1–80.8)	0.2862
Yes	64.8% (46.2–78.4)	
*Pre-operative Frankel score*		
E	67.5% (43.6–83.0	0.5780
other	56.7% (29.3–77.0)	
*Post-operative Frankel score*		
E	63.3% (44.4–77.3)	0.9992
other	58.3% (7.7–89.3)	
*Tokuhashi score*		
0–8	42.9% (9.8–73.4)	0.1523
9–11	61.9% (37.6–79.0)	
12–15	100%	
*Visceral Metastases*		
No	71.7% (48.7–85.7)	0.1747
Yes	55.8% (25.5–77.9)	
*Other metastases*		
No	77.7% (52.8–90.5)	0.0210
Yes	44.7% (19.5–67.3)	
*Complications*		
No	65.4% (43.9–80.4)	0.5916
Yes	55.9% (23.0–79.5)	
*Surgery types*		
Debulking	55.2% (34.3–71.8)	0.1180
En-bloc	100%	
other	66.7% (19.5–90.4)	
*Pre-radiation*		
No	80.4% (55.3–92.2)	0.0047
Yes	44.0% (18.0–67.5)	
*Post-radiation*		
No	62.9% (36.0–81.1)	0.6380
Yes	67.0% (39.6–84.2)	
*Pre-chemotherapy*		
No	73.9% (42.7–89.8)	0.1973
Yes	54.4% (28.1–74.7)	
*Post-chemotherapy*		
No	66.7% (5.4–94.5)	0.6755
Yes	65.1% (45.0–79.3)	

Note: nr, not reached.

## Data Availability

The data presented in this study are available on request from the corresponding author. The data are not publicly available due to privacy restrictions.
